# *HERV-K* and *LINE-1* DNA Methylation and Reexpression in Urothelial Carcinoma

**DOI:** 10.3389/fonc.2013.00255

**Published:** 2013-09-26

**Authors:** Ulrike Kreimer, Wolfgang A. Schulz, Annemarie Koch, Günter Niegisch, Wolfgang Goering

**Affiliations:** ^1^Department of Urology, Medical Faculty, Heinrich Heine University, Düsseldorf, Germany

**Keywords:** urothelial carcinoma, DNA hypomethylation, retroelements, *HERV-K*, *LINE-1*, *Alu*

## Abstract

Changes in DNA methylation frequently accompany cancer development. One prominent change is an apparently genome-wide decrease in methylcytosine that is often ascribed to DNA hypomethylation at retroelements comprising nearly half the genome. DNA hypomethylation may allow reactivation of retroelements, enabling retrotransposition, and causing gene expression disturbances favoring tumor development. However, neither the extent of hypomethylation nor of retroelement reactivation are precisely known. We therefore assessed DNA methylation and expression of three major classes of retroelements (*LINE-1*, *HERV-K*, and *AluY*) in human urinary bladder cancer tissues and cell lines by pyrosequencing and quantitative reverse transcription–polymerase chain reaction, respectively. We found substantial global *LINE-1* DNA hypomethylation in bladder cancer going along with a shift toward full-length *LINE-1* expression. Thus, pronounced differences in *LINE-1* expression were observed, which may be promoted, among others, by *LINE-1* hypomethylation. Significant DNA hypomethylation was found at the *HERV-K_22q11.23* proviral long terminal repeat (LTR) in bladder cancer tissues but without reactivation of its expression. DNA methylation of *HERVK17*, essentially absent from normal urothelial cells, was elevated in cell lines from invasive bladder cancers. Accordingly, the faint expression of *HERVK17* in normal urothelial cells disappeared in such cancer cell lines. Of 16 additional *HERV-Ks*, expression of 7 could be detected in the bladder, albeit generally at low levels. Unlike in prostate cancers, none of these showed significant expression changes in bladder cancer. In contrast, expression of the *AluYb8* but not of the *AluYa5* family was significantly increased in bladder cancer tissues. Collectively, our findings demonstrate a remarkable specificity of changes in expression and DNA methylation of retroelements in bladder cancer with a significantly different pattern from that in prostate cancer.

## Introduction

Human cancers are marked by several genetic and epigenetic changes. In particular, it is well established that aberrant DNA methylation contributes to cancer development. At specific loci, DNA hypermethylation in combination with the gain of a repressive chromatin conformation leads to gene silencing (1). Notably, whereas hypermethylation often targets genes already weakly transcribed in normal tissues, some epigenetically repressed genes exert tumor suppressive functions and their silencing by hypermethylation contributes to tumor development (2). In contrast to hypermethylation affecting single genes or selected genomic regions, a global decrease of methylcytosine occurs in cancers (3, 4). In benign tissues the bulk of the genome is densely DNA methylated. This methylation is believed to be one mechanism of restraining the activity of retroelements that comprise nearly half of the human genome (5). As several retroelements have retained their ability to retrotranspose, hypomethylation was proposed to enable their reactivation and thereby to contribute to the destabilization of the genome in cancer cells (4, 6). Global hypomethylation has been observed in many cancers, albeit occurring at different stages and to varying extents depending on the tumor entity (3).

For instance, many prostate cancers exhibit only a minor decrease in *LINE-1* promoter methylation (7) with hypomethylation aggravating in higher stage carcinomas (8). Conversely, urothelial carcinoma of the urinary bladder was reported to show a higher decrease of *LINE-1* methylation which was prevalent across all tumor stages (9–11). Likewise, hypomethylation of *HERV-K* retroelements was observed in bladder cancer cell lines and tissues correlating well with the decline of *LINE-1* methylation (9). However, activation of *HERV-K* elements was not found in that study. In prostate cancers one specific *HERV-K* element, referred to as *HERV-K_22q11.23* (*H22q*) was strikingly upregulated in cancerous tissues and this overexpression correlated well with hypomethylation of its long terminal repeat (LTR) (7). Remarkably, mRNA expression of the *H22q* provirus has been reported in several tumor entities and antibodies against H22q Gag protein have been detected in sera from bladder, liver, lung, ovarian, and prostate cancer patients (12). Interestingly, the androgen-responsive LTRs of *H22q* and *HERVK17* are involved in translocation events with *ETV1*, a member of the ETS family of transcription factors (13, 14). These events represent rare variants of the common translocations in prostate cancer that lead to oncogenic activation of ETS transcription factors.

While the above findings have highlighted the potential role of *HERV-K* proviruses in prostate carcinogenesis, reports concerning *HERV-K* expression in bladder cancer remain sporadic. By using massively parallel signature sequencing (MPSS) Stauffer et al. (15) detected *HERV-K* expression in normal bladder tissue resulting from a subgroup of 17 *HERV-K* elements. Of particular interest might be the expression of the melanoma-associated antigen HERV-K-MEL in a subset of bladder cancers (16). We have now conducted a broader and detailed analysis of retroelement DNA methylation and expression changes in urothelial carcinomas using mainly established quantitative pyrosequencing and quantitative reverse transcription PCR (qRT-PCR) methods previously applied to prostate cancer. This allows a direct comparison of methylation and expression changes between these genitourinary cancer entities.

## Materials and Methods

### Tissue samples and cell lines

Patients and tumor characteristics are compiled in Table 1. Patient consent was obtained and the study approved by the Ethics Committee of the Medical Faculty of the Heinrich Heine University. All urothelial cancer cell lines (253J, 5637, 639-V, 647-V, BFTC-905, HT-1376, J82, MGH-U4, RT4, RT-112, SCaBER, SD, SW1710, UMUC3, UMUC6, VMCUB1, T24) and cancer-associated fibroblasts were cultured in DMEM GlutaMax (Gibco, Darmstadt, Germany), supplemented with 10% fetal calf serum as described previously using standard methods (17). The cell lines were obtained from the DSMZ (Braunschweig, Germany), except UMUC3, kindly provided by Dr. Grossman, Houston. The telomerase-immortalized TERT-NHUC cell line was kindly provided by Prof. M. A. Knowles (Leeds, UK) and cultured as described previously (18). The well-differentiated urothelial carcinoma cell line BC61 established in our lab was cultured as described (19). Primary urothelial cells cultures (UP) were established from ureters after nephrectomy and were routinely maintained in keratinocyte serum-free medium (KSFM, Gibco, Darmstadt, Germany) supplemented with 12.5 μg/ml bovine pituitary extract and 0.25 ng/ml epidermal growth factor as described previously (20).

**Table 1:** **Clinical characterization of tissue sample sets**.

		DNA set (*n* = 23)	RNA set (*n* = 24)
Age	Median	65	66
	95% CI	61–71	63–69
	Range	41–84	45–84
Gender, *n* (%)	Female	7 (30.4)	5 (20.8)
	Male	16 (69.6)	19 (79.2)
Pathological T stage, *n* (%)	pTa	3 (13.0)	1 (4.2)
	pT1	1 (4.3)	0 (0)
	T2	4 (17.4)	7 (29.2)
	T3	10 (43.5)	11 (45.8)
	T4	5 (21.7)	5 (20.8)
Nodal status, *n* (%)	Negative	11 (47.8)	13 (54.2)
	Positive	7 (30.4)	8 (33.3)
	Unknown	5 (21.7)	3 (12.5)
Tumor grading, *n* (%)	G1	1 (4.3)	0 (0)
	G2	5 (21.7)	7 (29.2)
	G3	17 (73.9)	17 (70.8)

#### Nucleic acids extraction and quantitative reverse transcription–polymerase chain reaction

**Figure 1:**
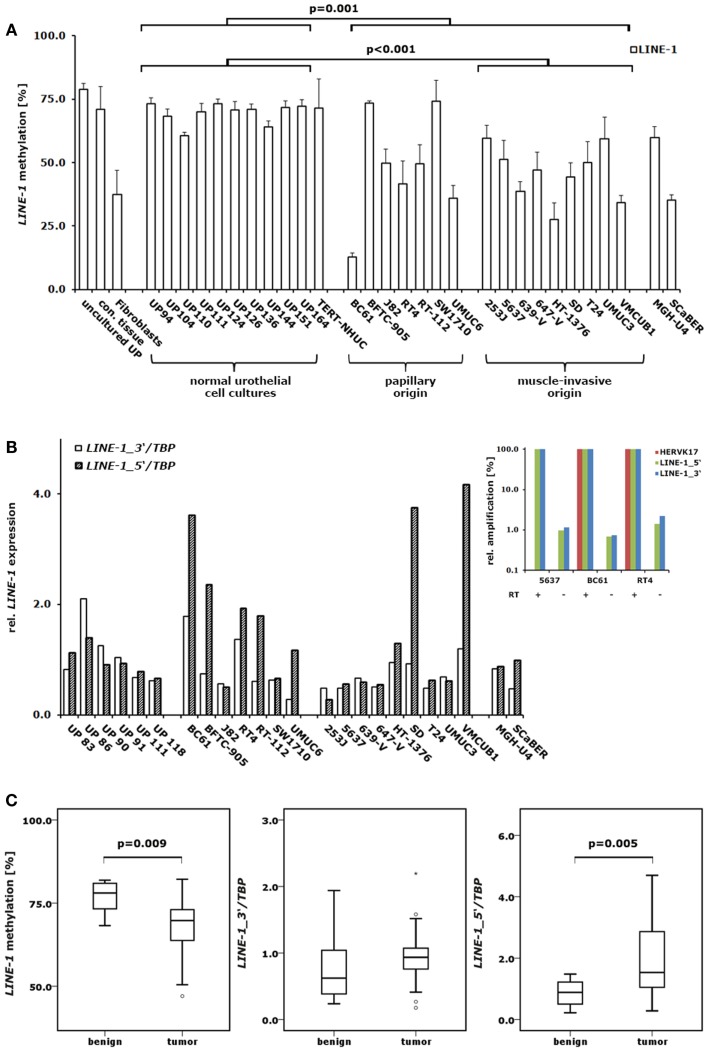
**DNA methylation and expression changes of *LINE-1* elements in bladder cancer**. **(A)** DNA methylation in the CpG islets of *LINE-1* was quantified by pyrosequencing in a set of 10 normal urothelial cell cultures and 18 bladder cancer cell lines. For comparison, *LINE-1* DNA methylation was assessed in immortalized urothelial cells (TERT-NHUC) and in uncultured epithelial cells (uncultured UP) and connective tissue from one ureter. **(B)** *LINE-1* RNA levels from the 5′- and 3′-regions were measured by qRT-PCR in a set of 6 normal urothelial cell cultures and 18 bladder cancer cell lines. Inset: amplification of different retroelements (*HERVK17*, *LINE-1_5*′ and *LINE-1_3*′) was measured in three bladder cancer cell lines using cDNA preparations with (+) or without (−) reverse transcriptase (RT) to assess the impact of genomic DNA contamination. Results were adjusted for each assay and cell line to reverse transcriptase positive preparations set as 100% **(C)** *LINE-1* DNA methylation and expression of the 5′- and 3′-regions were analyzed in a set of 12 benign and 23 cancerous bladder tissues or 11 benign and 24 tumorous bladder tissues, respectively. Methylation is plotted as mean methylation value from four CpGs in percent **(A,C)**. RNA levels were each normalized to *TBP* and standardized to either the median RNA level of normal urothelial cell cultures **(B)** or the median RNA level of benign bladder tissues **(C)** set as 1. *p* Values calculated by the Mann–Whitney *U*-test were given above the brackets for significant changes (*p* < 0.05). Missing *p* values demonstrate changes without reaching the level of significance.

High molecular weight DNA and total RNA were extracted from powdered tissues using standard protocols. Notably, RNA extraction involved acid phenol extraction followed by column purification to minimize DNA contamination. Further DNA contamination was removed by synthesis of complementary DNA including a DNA removal step by DNase using the QuantiTect Reverse Transcription Kit (Qiagen, Hilden, Germany), according to the manufacturer’s protocol. In order to estimate the remaining levels of genomic DNA after cDNA preparation, amplification values for three different retroelement specific qPCR assays (*HERVK17*, *LINE-1_5′* and *LINE-1_3′*) were assessed by quantitative PCR using cDNA preparations from three different bladder cancer cell lines (5637, BC61 and RT4) with or without reverse transcriptase (RT) treatment after DNA removal. As shown in Figure 1B (inset), amplification levels of background genomic DNA were at most around 1% of the total expression of high-copy retroelements (*LINE-1_5′* and *LINE-1_3′*). With an assay for single-copy retroelement (*HERVK17*) amplification from genomic DNA was essential absent (cf. Figure 1B).

Quantitative reverse transcription (qRT)–PCR was performed as described previously (7) on a 7500 Fast Real-Time PCR System (Applied Biosystems, Carlsbad, CA, USA) using QuantiTect SYBR Green PCR Kit (Qiagen). Initial qualitative PCR with specific primers listed in Table 2 was performed as following: initial denaturation step at 95°C for 15 min, followed by 35 amplification cycles consisting of denaturation at 94°C for 15 s, annealing at 56°C for 15 s, and extension at 72°C for 30 s. Samples were analyzed on 1.5% agarose gels. Assays with detectable transcripts in this qualitative PCR were subjected to quantitative PCR analysis. All qRT-PCR data were adjusted to *TATA-box-binding protein* (*TBP*) mRNA measured by a specific *TBP* assay (Table 2). For all other transcripts, specifically designed primers (Table 2) were employed using the following PCR conditions: initial denaturation step at 95°C for 15 min, followed by 40 amplification cycles consisting of denaturation at 95°C for 15 s, annealing for 20 s, and extension at 72°C for 30 s. All measurements were performed in at least duplicates; assay variance was <10%. Relative expression was calculated by a modified ΔΔCt method published by Pfaffl (21).

**Table 2:** **Oligonucleotides**.

Gene/region	Sequence	Bases (NCBI/hg19)	Primer	Sequence 5′–3′
HERV-K_3p25.3	Chromosome 3	9,895,774–9,895,791	H3p25_for	GCATCTGTCTCCTGCTTG
HERV-K_3p25.3	Chromosome 3	9,895,722–9,895,740	H3p25_rev	ATCTCAGTAGATGGAATCG
HERV-K_3q12.3	Chromosome 3	101,411,166–101,411,184	H3q12_for	GTGCTGAGGAGGATTAGTG
HERV-K_3q12.3	Chromosome 3	101,411,341–101,411,358	H3q12_rev	AGTATTGCTGCCGGCTTG
HERV-K_3q21.2	Chromosome 3	125,609,734–125,609,755	H3q21_for	ATTAGTAAAAGAGGAAAGAATG
HERV-K_3q21.2	Chromosome 3	125,609,821-125,609,842	H3q21_rev	CATACAATCAGGTTTTATACTG
HERV-K_3q27.2	Chromosome 3	185,288,247–185,288,266	H3q27_for	CATGGTTTCCAGAACAAGAA
HERV-K_3q27.2	Chromosome 3	185,288,095–185,288,116	H3q27_rev	GAAACTGAAACGCTATCTTCTG
HERV-K_10p14	Chromosome 10	6,875,319–6,875,337	H10p14_for	CTCAACTACCCAGGGATAC
HERV-K_10p14	Chromosome 10	6,875,183–6,875,200	H10p14_rev	TTACGGGTGTCGAGCTGC
HERV-K_11q22.1	Chromosome 11	101,572,720–101,572,739	H11q22_for	TCCTATTTGCTTAGGGACAG
HERV-K_11q22.1	Chromosome 11	101,572,768–101,572,787	H11q22_rev	GTACTTCTACCAACCAGTTT
HERV-K_12q14.1	Chromosome 12	58,730,155–58,730,176	H12q14_for	TGTCTCGGTATAAAACCTGACT
HERV-K_12q14.1	Chromosome 12	58,729,973–58,729,993	H12q14_rev	GTCAGCAGACAAACATGTGAA
HERV-K_12q24.11	Chromosome 12	111,008,880–111,008,897	H12q24_for	GACGAGAGATCCCGAGGA
HERV-K_12q24.11	Chromosome 12	111,008,929–111,008,947	H12q24_rev	CCCTAGCTTCTTCCGAGTG
HERV-K_10	Chromosome 5	156,085,657–156,085,678	H10_for	GAAAAGCAAGAGAGATCAAATT
HERV-K_10	Chromosome 5	156,085,583–156,085,603	H10_rev	GCAGAAGAATTTTTCTTAGCA
HERV-K_18.2	Chromosome 1	160,661,401–160,661,422	H18_for	ATCCTCCATATGCTGAACGTTG
HERV-K_18.2	Chromosome 1	160,661,489–160,661,510	H18_rev	TGTTTCTCGTAAGGTGCAATGA
HERV-K_102	AF164610.1 (NCBI)	819–836	H102_for	TGGCGGGATCCTCCACAT
HERV-K_102	AF164610.1 (NCBI)	910–928	H102_rev	CGTAAGGTGGGATGAGAGA
HERV-K_104	AF164612.1 (NCBI)	239–295	H104_for	AGTCATCACCACTCCCTCATC
HERV-K_104	AF164612.1 (NCBI)	333–355	H104_rev	GCCATATTTCAGACTATGAAACC
HERV-K_108	AF164614.1 (NCBI)	778–795	H108_for	CACCCACAGATGATCAGT
HERV-K_108	AF164614.1 (NCBI)	908–925	H108_rev	AAGGTGGGACGAGAGATT
HERV-K_113	AY037928.1 (NCBI)	565–582	H113_for	TAGGGAAAAACCGCCTCA
HERV-K_113	AY037928.1 (NCBI)	694–711	H113_rev	CGTGAACAAAGGTCTTGG
TBP	CR456776.1 (NCBI)	11–29	TBP_for	ACAACAGCCTGCCACCTTA
TBP	CR456776.1 (NCBI)	111–130	TBP_rev	GAATAGGCTGTGGGGTCAGT

Primer assays for expression analysis of AluYa5, AluYb8, H22q, HERVK17, HERV-K_11q23.3, HERV-K_22q11.21, LINE-1_5′, LINE-1_3′ and pyrosequencing assays for H22q, HERVK17, LINE-1 were published previously (7).

### Bisulfite treatment and DNA methylation analyses

Bisulfite conversion was performed using the EZ DNA Methylation-Gold Kit (Zymo Research, Hiss Diagnostics, Freiburg, Germany) according to the manufacturer’s instructions. Bisulfite-treated DNA samples were used for PCR with the indicated primers (Table 2) using HotStartTaq (Qiagen) under the following conditions: initial denaturation step at 95°C for 15 min, followed by 45 amplification cycles consisting of denaturation at 95°C for 15 s, annealing for 20 s, and extension at 72°C for 45 s. Pyrosequencing was carried out on a PyroMark Q24 instrument (Qiagen) according to the manufacturer’s instruction. Corresponding sequencing primers are listed in Table 2. Methylation values were calculated as the mean value of 4 (*LINE-1*) or 6 (*H22q*; *HERVK17*) CpG sites, respectively.

### Statistics

*p* Values were calculated by the Mann–Whitney *U-*test using SPSS Statistics 20 (IBM) software. Correlation coefficients and their respective level of significance were calculated by non-parametric Spearman’s rank correlation coefficient (Spearman’s ρ) using SPSS Statistics 20 (IBM) software.

## Results

### *LINE-1* DNA methylation and expression in urothelial cell cultures and cell lines

*LINE-1* promoter DNA methylation was assessed in a set of 10 primary urothelial cell cultures and 18 bladder cancer cell lines by pyrosequencing of bisulfite-treated DNA using an assay described previously (7). Briefly, the assay interrogates four CpG sites within the internal promoter of approximately 400 full-length *LINE-1* elements distributed across the genome. The bladder cancer cell lines are all derived from urothelial carcinomas with the exception of SCaBER (squamous cell carcinoma of the urinary bladder) and MGH-U4 (urothelial dysplasia). Additionally, bisulfite-treated DNA from bladder cancer-associated fibroblasts, immortalized urothelial cells (TERT-NHUC), and connective tissue originating from a freshly dissected ureter were used for comparison.

While primary normal urothelial cell cultures exhibited the high and homogenous *LINE-1* promoter methylation expected of normal cells, bladder cancer cell lines showed heterogeneous changes, but with overall significantly decreased methylation levels (Mann–Whitney *U*-test; *p* = 0.001) (Figure 1A). By grouping bladder carcinoma cell lines into those either originating from papillary (*n* = 7) or muscle-invasive (*n* = 9) cancers we found those with muscle-invasive cancer origin displaying more significant *LINE-1* hypomethylation compared to cultured normal urothelial cells (Mann–Whitney *U*-test; *p* < 0.001) (Figure 1A). Notably, the well-differentiated papillary cancer cell line BC61 showed the most prominent hypomethylation displaying only 14% mean *LINE-1* promoter DNA methylation. Conversely, the bladder papillary cell lines BFTC-905 and SW1710 retained high methylation at *LINE-1* promoters comparable with the levels in normal urothelial cells (Figure 1A). Immortalized urothelial cells (TERT-NHUC), uncultured epithelial cells and cells from connective ureter tissue exhibited the same *LINE-1* methylation levels found in urothelial cell cultures, whereas cancer-associated fibroblasts had comparably low methylation.

Expression analysis of *LINE-1* elements was performed on a set of 6 primary urothelial cell cultures and 18 bladder cancer cell lines from different origins (7 papillary; 9 muscle-invasive, 2 others) using two assays described previously (7) that detect either unspliced, full-length *LINE-1* transcripts (*LINE-1_5*′; ∼400 elements), or spliced and unspliced *LINE-1* transcripts (*LINE-1_3*′; ∼2000 elements). The *LINE-1_3*′-assay revealed decreased median transcript levels in bladder cancer cells compared to cultured normal urothelial cells but the changes were overall not significant (Figure 1B). In contrast, several cell lines showed increased expression by the *LINE-1_5*′-assay. Accordingly, we detected a prominent shift toward unspliced, full-length *LINE-1* transcripts in several bladder cancer cell lines. The bladder cancer cell lines BC61, BFTC-905, RT-112, UMUC6, SD, and VMCUB1 exhibited 2.0- to 4.2-fold higher normalized *LINE-1_5*′ transcript levels compared to the respective *LINE-1_3*′ mRNA levels. However, this shift was not found across all cell lines and was therefore not overall significant.

Correlation analyses of the *LINE-1* expression detected a robust and significant positive correlation between the two assessed *LINE-1* transcript variants in bladder cancer cell lines (Spearman’s ρ = 0.628; *p* = 0.005). In bladder cancer cell lines, *LINE-1* transcription correlated inversely with *LINE-1* DNA methylation without reaching the level of significance. Of note, inverse correlation of *LINE-1* DNA methylation with expression measured by the 5′-assay (Spearman’s ρ = 0.443; *p* = 0.066) was substantially better than that with the 3′-assay (Spearman’s ρ = 0.255; *p* = 0.307).

### *LINE-1* DNA methylation and expression in benign and bladder cancer tissues

To analyze *LINE-1* promoter DNA methylation and *LINE-1* transcript expression in benign and cancerous bladder tissues we performed methylation and expression analyses using our established pyrosequencing and quantitative RT-PCR assays on a set of 12 benign and 23 tumor probes and 11 benign and 24 cancer samples, respectively. Unfortunately, the DNA and RNA samples came from different studies with only limited overlap. *LINE-1* promoter DNA methylation was highly significantly decreased in bladder cancer specimen (Mann–Whitney *U*-test; *p* = 0.009) compared to normal tissues with striking differences in their percent median values (Δ_median_ = 8.5%) (Figure 1C). Like the decrease in DNA methylation, *LINE-1* expression changes were also similar in bladder tumor tissues to those found in cultured cells. The median levels of transcripts assessed by the *LINE-1_3*′-assay tended to be slightly higher in bladder cancer specimen, but the changes were not significant (Mann–Whitney *U*-test; *p* = 0.123) (Figure 1C). In contrast, analyses of full-length *LINE-1* transcripts using the *LINE-1_5*′-assay revealed a significant increase of full-length transcript levels in bladder tumor tissues (Mann–Whitney *U*-test; *p* = 0.005) (Figure 1C). Taken together, these changes resulted in a shift toward full-length *LINE-1* expression. Due to the limited overlap of DNA and RNA samples the analysis of the correlation between DNA methylation and expression was not possible.

### *AluYa5* and *AluYb8* expression in benign and bladder cancer samples

**Figure 2:**
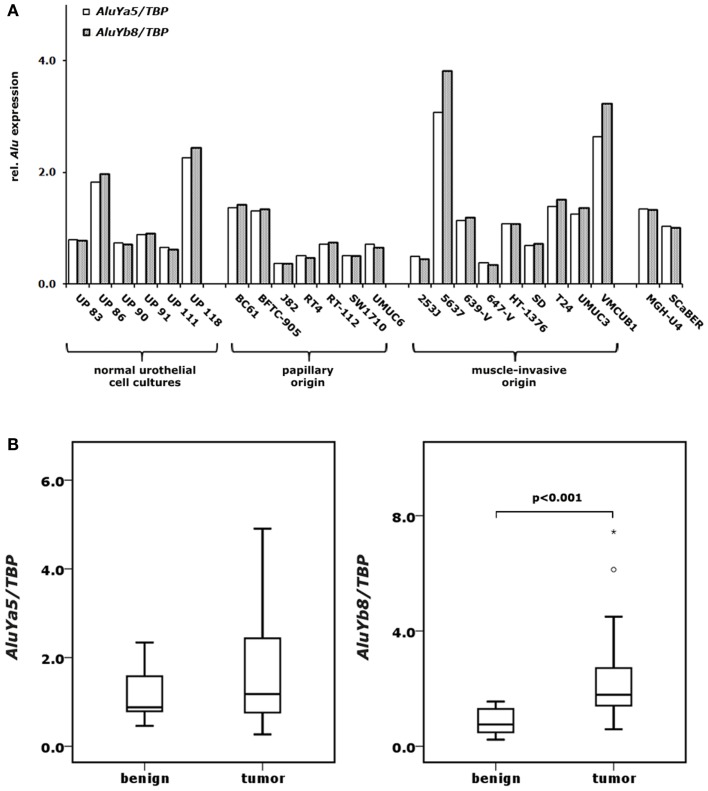
**Expression changes of *AluYa5* and *AluYb8* in bladder cancer**. *AluYa5* and *AluYb8* RNA levels were measured by qRT-PCR in 6 normal urothelial cell cultures and 18 bladder cancer cell lines **(A)** as well as in 11 benign and 24 bladder cancer samples **(B)**. RNA levels were each normalized to *TBP* and standardized to either the median RNA level of normal urothelial cell cultures **(A)** or the median RNA level of benign bladder tissues **(B)** set as 1. *p* Values calculated by the Mann–Whitney *U*-test were given above the brackets for significant changes (*p* < 0.05). Missing *p* values demonstrate changes without reaching the level of significance.

Additionally, we investigated the expression of the two most commonly active retroelements of the *AluY* family (*AluYa5* and *AluYb8*) in our set of primary urothelial cell cultures and bladder cancer cell lines. We found robust expression of both elements in the primary urothelial cell cultures (Figure 2A). The expression of both elements tended to be diminished in cancer cell lines of papillary origin and was slightly increased in cell lines from muscle-invasive carcinomas without the difference reaching the level of significance (Figure 2A). Of note, the expression of both elements correlated strikingly throughout all samples (Spearman’s ρ = 0.990; *p* < 0.001). By applying the same assays to our set of benign and bladder cancer tissues we found no significant changes in the expression of the *AluYa5* retroelements. Instead, *AluYb8* transcript levels were highly significantly increased in bladder cancer specimens (Mann–Whitney *U*-test; *p* < 0.001) (Figure 2B). Other than in the cell lines, RNA levels of *AluYb8* showed only a weaker and non-significant correlation to *AluYa5* expression in the cancer tissues (Spearman’s ρ = 0.354; *p* = 0.090).

### DNA methylation of *HERV-K* LTRs in benign and bladder cancer probes

**Figure 3:**
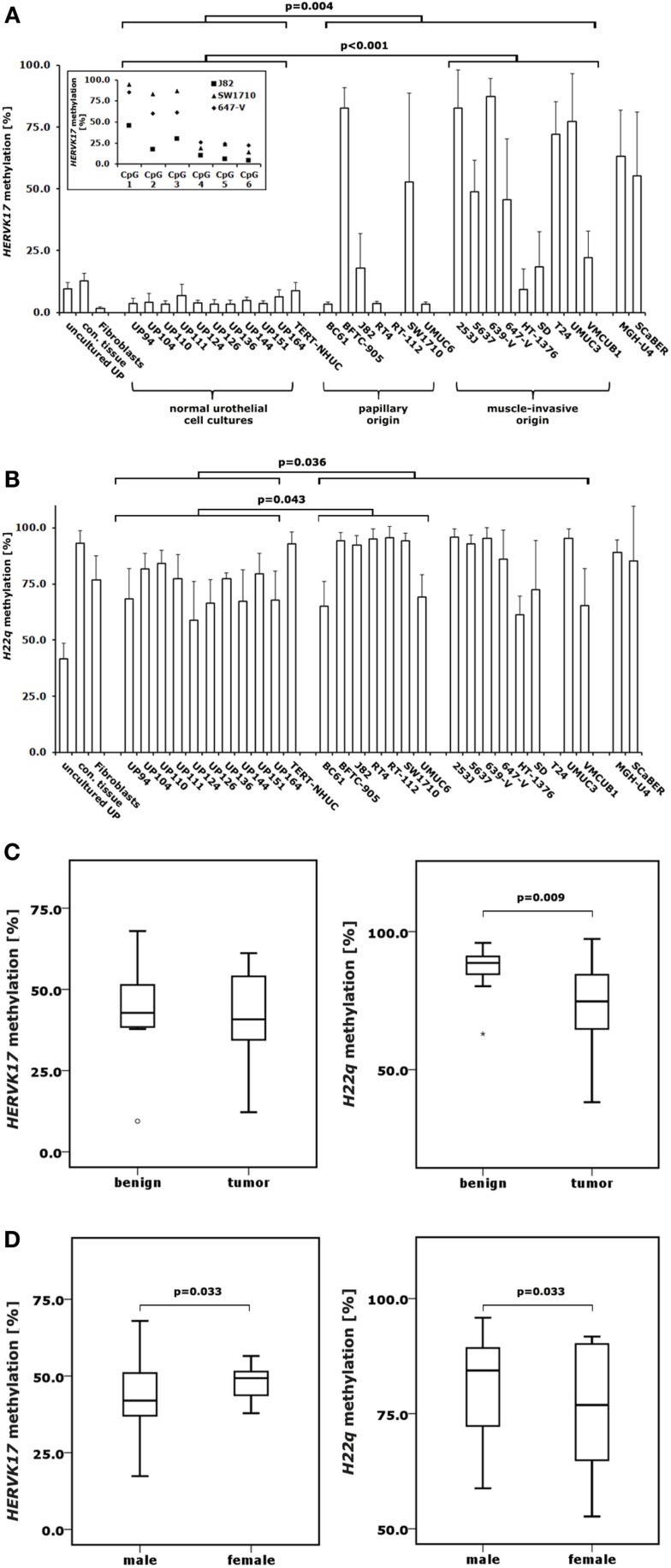
**DNA methylation changes in proviral *HERVK17* and *H22q* LTRs in bladder cancer**. DNA methylation in the LTRs of *HERVK17* **(A)** and *H22q* **(B)** were analyzed by pyrosequencing in 10 normal urothelial cell cultures and 18 bladder cancer cell lines. Additionally, *HERVK17* and *H22q* DNA methylation was assessed in immortalized urothelial cells (TERT-NHUC) and in uncultured epithelial cells (uncultured UP) and connective tissue from one ureter. **(C)** DNA methylation of *HERVK17* and *H22q* LTRs were each analyzed in a set of 12 benign and 23 cancerous bladder tissues. **(D)** DNA methylation of *HERVK17* and *H22q* LTRs from tumor samples were each plotted against patients’ gender. Methylation is plotted as mean methylation value from six CpGs each in percent. The high standard deviation in some samples results from differences in the methylation within the *HERVK17* sequence, where the first three CpGs are generally higher methylated as exemplified for data from J82, SW1710, and 647-V bladder cancer cell lines in the insert **(A)**. *p* Values calculated by the Mann–Whitney *U*-test are given above the brackets for significant changes (*p* < 0.05). Missing *p* values demonstrate changes without reaching the level of significance.

In order to investigate DNA methylation at *HERV-K* LTRs in urothelial samples, we used two previously established pyrosequencing assays to analyze *HERVK17* and *H22q* methylation in bisulfite-converted DNA samples from the normal urothelial cell cultures, bladder cancer cell lines, benign and bladder cancer tissues also investigated for *LINE-1* methylation. Intriguingly, we found the *HERVK17* LTR to be essentially demethylated in normal urothelial cell cultures, but becoming hypermethylated in bladder cancer cells (Figure 3A). Noteworthy, DNA methylation levels in the *HERVK17* LTR remained low in most bladder cell lines of papillary origin with no significant changes compared to cultured urothelial cells (Mann–Whitney *U*-test; *p* = 0.635). Significantly elevated *HERVK17* DNA methylation values were instead regularly found in cancer cells derived from muscle-invasive bladder carcinomas (Mann–Whitney *U*-test; *p* < 0.001) (Figure 3A). Interestingly, *HERVK17* LTR methylation was considerably higher in normal bladder tissues (median: 44.9%) compared to normal urothelial cell cultures (median: 3.7%), remaining on the same level in bladder cancer tissues (Figures 3A,C).

DNA methylation of the *H22q* proviral LTR was high in benign bladder tissues (88.6%) and declined significantly in bladder cancer specimens (72.4%, Mann–Whitney *U*-test; *p* = 0.009) (Figure 3C). Overall, LTR DNA methylation of both *HERV-K* proviruses correlated well and highly significantly (Spearman’s ρ = 0.669; *p* < 0.001) in bladder cancer tissues. Although overall comparable DNA methylation changes were found for *H22q* and *LINE-1* no correlation was detectable. Unexpectedly, the *H22q* provirus was not hypomethylated, but significantly hypermethylated in bladder cancer cell lines compared to cultured normal urothelial cells (Mann–Whitney *U*-test; *p* = 0.036) (Figure 3B). Additionally, hypermethylation of the *H22q* LTR was more prominent in papillary cancer cell lines.

DNA methylation of *H22q* LTR was slightly but significantly lower in bladder cancer tissues originating from female patients (Mann–Whitney *U*-test; *p* = 0.033) (Figure 3D). Conversely, LTR methylation of the *HERVK17* provirus was significantly higher in female cancers (Mann–Whitney *U*-test; *p* = 0.033). In contrast, *LINE-1* promoter methylation showed no significant gender-specific differences in cancers.

### Expression analyses of different *HERV-K* proviruses

**Figure 4:**
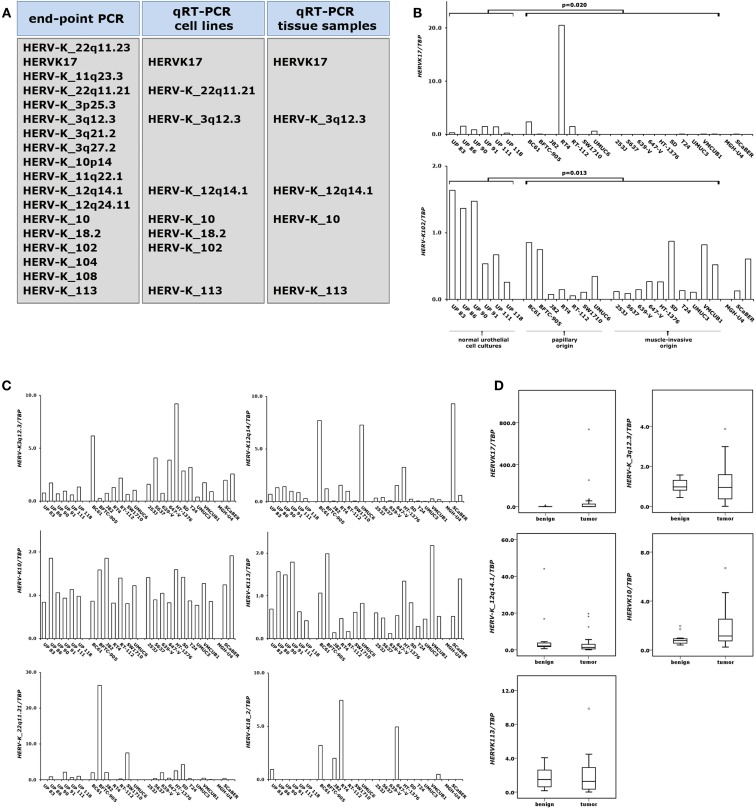
**Expression changes of proviral *HERV-K* elements in bladder cancer**. Expression of different *HERV-K* elements was assessed by end-point PCR and qRT-PCR as indicated in **(A)**. *HERV-K* RNA levels were measured by qRT-PCR in 6 normal urothelial cell cultures and 18 bladder cancer cell lines **(B,C)** and in a set of 11 benign and 24 cancerous bladder tissues **(D)**. *p* Values calculated by the Mann–Whitney *U*-test were given above the brackets for significant changes (*p* < 0.05). Missing *p* values demonstrate changes without reaching the level of significance.

To assess *HERV-K* expression in benign and cancerous urothelial samples we conducted qRT-PCR analyses on our set of normal urothelial cell cultures, bladder cancer cell lines, benign and bladder tumor tissues. Initially, we performed expression analyses of the four *HERV-K* retroelements which had previously been investigated in prostate samples by our group (7). Then, we established qRT-PCR assays for 14 additional *HERV-K* elements which had been described as possibly expressed in bladder tissue by using massively parallel signature sequencing (MPSS) (15). The strategy for analysis of the expression of these *HERV-K* elements is illustrated in Figure 4A. First, we performed standard end-point PCR on our set of cultured normal urothelial and bladder cancer cells. Transcriptionally active *HERV-K* elements were subjected to quantitative RT-PCR using the same sample set. Those *HERV-K* elements exhibiting detectable RNA levels in normal cultured and urothelial cancer cells were analyzed for their expression in benign and cancerous bladder tissues. We then conducted qRT-PCR analyses on the eight *HERV-K* elements detectable in our set of normal urothelial cell cultures and bladder cancer cell line.

In general, expression of these *HERV-K* elements was rather low in these samples bordering on the limit of reliable quantification (Figures 4B,C). Two of the analyzed *HERV-K* elements (*HERVK17* and *HERV-K102*) showed significant expression changes between normal urothelial cell cultures and bladder cancer cell lines. *HERV-K102* was significantly downregulated (Mann–Whitney *U*-test; *p* = 0.013) in bladder cancer cells independent of the tumor type of origin, but expression was on the limit of detection (Figure 4B). In general, expression of the *HERVK17* provirus was downregulated as well. In cultured normal urothelial cells its transcript level was low and these low expression levels were preserved in most papillary carcinoma cell lines (Figure 4B). Exceptionally, the RT4 cell line showed a high increase of *HERVK17* expression. Notably, all papillary cell lines positive for *HERVK17* expression (BC61, RT4, and UMUC6) (Figure 4B) showed low methylation levels at the *HERVK17* LTR comparable to those found in cultured normal urothelial cells (Figure 3A). In cell lines originating from muscle-invasive bladder carcinoma *HERVK17* expression was essentially absent (Figure 4B) fitting well with the hypermethylation found at the *HERVK17* locus in the respective cell lines (Figure 3A). Expression of the other *HERV-K* elements was low and no significant expression changes were observed in bladder cancer cell lines (Figure 4C).

In benign bladder samples expression of the *HERVK17* provirus was low or absent with one exception showing significant expression (Figure 4D). Likewise, most of the bladder cancers exhibited low or absent expression of the *HERVK17* locus, whereas a few samples showed strikingly increased expression (up to 700-fold). Across all samples, the expression of the *HERVK17* provirus was not significantly changed (Mann–Whitney *U*-test; *p* = 0.687). Expression levels of the other *HERV-K* retroelements (*HERV-K10*, *HERV-K_3q12.3*, *HERV-K_12q14.1*, *HERV-K113*) assessed in our bladder tissue set were rather low and no significant expression increases were found in cancerous tissues (Figure 4D).

## Discussion

In the present study we describe the impact of global methylation changes in bladder cancers tissues and cell lines on the most important classes of active retroelements in the human genome. With respect to *LINE-1* sequences, which make up 17% of the genome, the quantitative methylation data obtained in this study confirm previous finding of widespread hypomethylation in bladder cancers (9–11). Results of the DNA methylation analyses in bladder cancer cell lines revealed a tendency toward exacerbation in high-grade and high-stage tumors, but also changes in cell lines from papillary tumors. Evidently, *LINE-1* hypomethylation is an early and very frequent change in bladder cancer. Quantitative changes of *LINE-1* methylation were comparable to those in colorectal cancers where *LINE-1* hypomethylation also occurs early, but are more pronounced compared to those in prostate cancer where *LINE-1* hypomethylation is a later event during carcinogenesis (7, 8, 22–24).

Nevertheless, the hypomethylation of the *LINE-1* promoter found in bladder cancer cell lines did not result in overall increased *LINE-1* expression, but went along with a shift toward full-length *LINE-1* expression as previously observed in prostate cancers (7). A previous report has described the spliced form of *LINE-1* transcripts as the predominant isoform in normal prostate tissue (25), which also seems to be the case in the urinary bladder. In bladder cancer tissues the more prominent hypomethylation compared to prostate cancer went along with a more general increase in *LINE-1* expression but only the expression of the full-length *LINE-1* isoform increased significantly. Notably, in bladder cancer cell lines the correlation of *LINE-1* promoter methylation with the expression of the full-length transcript assessed by the 5′-assay was better than the correlation with the 3′-assay. Taken together, the evidence suggests that the hypomethylation of the *LINE-1* promoter in cancers may contribute to a shift toward full-length *LINE-1* expression. The most obvious explanation for that observation is that most *LINE-1* transcripts in normal tissues originate from truncated elements that represent parts of longer transcripts from host gene promoters, whereas hypomethylation in cancer may allow a degree of transcription initiation from *LINE-1* 5′-internal promoters. In addition, *LINE-1* promoter DNA hypomethylation may enable expression from the *LINE-1* antisense promoter (ASP) resulting in cancer-specific chimeric transcripts (26). Noteworthy, DNA hypomethylation of a specific LINE-1 element within the *MET* oncogene was recently shown to enable expression of a chimeric *L1-MET* transcript starting from the ASP in bladder cancers (10). As the *LINE-1* ASP is located in the *L1Hs* 5′-UTR between nucleotides 400–600 (27), transcripts starting from the ASP are detectable by our *LINE-1_5′*-assay. Hence, elevated expression detected by the *LINE-1_5′* assay may partially reflect *LINE-1* ASP activation.

Whereas *TP53* mutations are relatively infrequent in prostate carcinoma, in invasive bladder cancers *TP53* missense mutations are found in around 50% of the cases. Additional alterations of “upstream” or “downstream” factors within the p53 network contributing to p53 inhibition are common (28). Accordingly, the more prominent impairment of *LINE-1* expression in bladder cancers compared to prostate cancers may be partially explained by p53 mediated regulation of *LINE-1* expression (29, 30).

Notably, only full-length *LINE-1* transcripts contain information for both *LINE-1* open reading frames (ORF1 and ORF2) required for retrotransposition of *LINE-1* (31, 32) and other non-autonomous retroelements like SINEs and processed pseudogenes. Up to now roughly 100 disease-causing retrotransposition events, usually occurring during early embryonic or germ line development, are known, predominantly elicited by retrotransposed *AluY* elements (33). Additionally, within the last few years several reports described examples of *LINE-1* retrotransposition in different cancers (34–37). Analyses for bladder cancers are still missing, but will presumably be enabled soon by the data from an ongoing whole-genome sequencing project.

Surprisingly, in contrast to the hypomethylation observed at the *LINE-1* promoter in bladder cancer cell lines as compared to normal urothelial cells the LTRs of *HERVK17* and *H22q* showed overall significantly increased methylation levels. Nevertheless, expression of the *H22q* provirus remained undetectable in any tumor. This is in good accordance to the study by Stauffer et al. (15). Here, *H22q* expression was not detectable by MPSS in bladder, but only in prostate samples. Predominant prostatic expression of this provirus was confirmed by others (7, 12, 13). In contrast, antibodies reacting with H22q Gag protein were found in the sera from bladder cancer patients (12). As in the same study *H22q* mRNA was not found in bladder carcinoma specimen, the positive antibody reaction may be due to cross-reactivity of a serum antibody to a different protein resembling the H22q Gag. *HERVK17* showed expression only in bladder cancer cell lines of papillary origin whereas expression of the provirus was nearly absent in muscle-invasive cell lines. Noteworthy, expression was only detectable in cell lines with low *HERVK17* methylation suggesting that DNA methylation may constitute one factor limiting its expression.

Many studies published in the last decade emphasize the strongly tissue- and cancer-specific expression pattern of *HERV-K* elements (7, 12, 38–41). The mechanisms underlying this pattern are still poorly understood, although tissue-specific transcription factors and epigenetic regulation are clearly implicated. In our present study expression of eight specific *HERV-K*s was detectable in urothelial cells by end-point PCR, whereas that of nine others was not. Quantification of these *HERV-K* transcript levels revealed generally low expression in normal bladder which is in good concordance to previously published results assessed by MPSS (15). Among the faintly expressed elements was the *HERV-K113* provirus. Its expression in nearly all bladder samples does not fit with previous observations that *HERV-K113* occurs in a small part of the human population. *HERV-K113* was likely acquired in Africa during or after the migration by *Homo sapiens* north and eastward and showed the highest frequencies in individuals from central Africa (∼20–30%) (42, 43). A large study assessing more than 2800 individuals in the UK found *HERV-K113* allele frequency of approximately 7% (44). Most likely, the weak *HERV-K113* expression in our data was at least partially caused by cross-reactivity of the used assay with another very closely related *HERV-K* element. Except for *HERV-K102* and *HERVK17* (as discussed above) significant cancer-specific expression changes of these elements were detectable neither in bladder cancer cell lines nor tissues. Transcripts of the proviruses *HERV-K_11q23.3* and *HERV-K_22q11.21* are strongly expressed in testicular cancers (38) but not in prostate tissues (7). Of these, only *HERV-K_22q11.21* showed detectable expression in bladder tissues underlining again the strongly tissue-specific expression of distinct *HERV-K* elements.

In contrast to the methylation changes in bladder cancer cell lines *HERVK17* LTR methylation was decreased in bladder tumor tissues with a good correlation to *H22q* methylation changes. Puzzlingly, *HERVK17* LTR exhibited significant higher methylation in normal bladder tissues compared to cultured urothelial cells. In order to exclude that the LTR becomes demethylated during culture, we analyzed freshly prepared, uncultured urothelial cells, which showed only slightly higher methylation than the cultured cells. In addition, residual connective tissue from a ureter after removal of the epithelial layer also exhibited lower *HERK17* DNA methylation than benign bladder tissues. Instead, the *HERVK17* mean methylation value in benign bladder tissue is rather comparable to that found in benign prostate tissues [mean = 35.1 vs. 44.9%; (7)]. The difference toward cultured cells could therefore result from an admixture of other cell types, such as infiltrating immune cells that are prominent in cancer-carrying bladders or may reflect one of the few distinctive differences between ureter and bladder urothelium tissue (45).

DNA methylation of both *HERV-K* LTRs but not of the *LINE-1* promoter showed significant correlation to patient gender. One explanation for these differences may be the well-known influence of androgens on the development of bladder cancer (46). The correlation could also result from a higher fraction of smokers in the male population. Smoking is a main risk factor for urothelial cancers accounting partly for its higher incidence in males (47). As smoking induces several epigenetic changes in urothelial cells (48) it may also affect *HERV* methylation and contribute to aberrant *HERV* expression. Additionally, *HERVs* were reported to become induced by smoking in urothelial cell lines and tissues (49) which may be causative for *HERVK17* expression in a few cancer tissues. As the smoking status was not consistently assessed in our patient cohort we cannot confirm these assumptions.

To unravel the puzzle of the regulation of specific *HERV* elements high-throughput transcript analyses of *HERV* expression are highly desirable. Likewise, detailed studies are needed to investigate the tissues-specific regulators of *HERV* expression as published by us and others (7, 50–53). In this respect, the present study provides a framework for studies on urothelial tissue.

Expression of *AluYa5* and *AluYb8* SINEs was not significantly altered in bladder cancer cell lines. In contrast, in bladder cancer tissues *AluYb8* but not *AluYa5* expression was highly significantly elevated. It is generally assumed that *Alu* induction is related to several different kinds of cellular stresses (54). Human *Alu* and rodent B2 SINE are activated in response to heat shock (55, 56) and are consecutively involved in heat shock-related stress response (57). *Alu* expression was elevated during hypoxic stress in human glioblastoma cells, whereas tRNA genes and B2 elements were inhibited in response to hypoxia in rat cardiomyocytes, even though tRNA genes and SINEs have very similar promoters (58, 59). As standardized cell culture conditions are unlikely to induce heat shock or hypoxic stresses, it is plausible to assume that only basal level of *Alu* transcription were observed in cultured cells. In bladder cancer tissues, a likely inducer of *AluYb8* expression is hypoxic stress as hypoxia is a well-known feature in this solid tumor (60). In contrast, *AluYa5* expression was only slightly altered and may not respond to this kind of cellular stress. The factors regulating SINE expression in stressed cells and the reasons why these factors do not affect the transcription of other small RNAs with comparable promoters are largely unknown (54). Moreover, our data hint at an element-specific regulation of *Alu* expression in response to cellular stresses. *Alu* elements are characterized by their huge number with limited diversity (61), which complicates methylation analyses and calls for genome-wide high-throughput approaches. Recently, such global sequencing approaches for *Alu* methylation analyses have revealed tissue-specific methylation of certain *Alu* elements (62) and a decline of *Alu* DNA methylation in several cancers which was most pronounced for members of the *AluY* family (63–66). In benign tissues the methylation level of specific *Alu* elements and the degree of their methylation heterogeneity is dependent on their genomic location and their adjacent sequence motifs and heterogeneity increases in cancer tissues (67). Interestingly, recent whole-genome sequencing studies suggest that besides *LINE-1*, retrotranspositions in human cancers substantially involve *Alu* elements (35). In that respect, our study invites the speculation that *LINE-1* activation in bladder cancers may also facilitate *AluYb8* retrotransposition in particular.

In summary, our study provides the first survey of expression and methylation of the most active retroelement classes in bladder cancer. Even though changes in *LINE-1* methylation and expression are more pronounced in this cancer type than those observed in our previous study on prostate cancer (7), the results further support our conclusion that apparently global DNA hypomethylation affects retroelements to very different extents and does not lead to wholesale reexpression. Rather, changes in methylation and expression may have to be investigated at many individual elements to identify those that contribute to genomic instability and deregulation of gene expression in each cancer type.

## Conflict of Interest Statement

The authors declare that the research was conducted in the absence of any commercial or financial relationships that could be construed as a potential conflict of interest.
